# The association of urinary xenoestrogens levels in pregnant women and their risk of missed miscarriage

**DOI:** 10.3389/fpubh.2026.1858374

**Published:** 2026-09-30

**Authors:** Tao Xu, Yanlu Yu, Xing Peng, Liming Gou, Yuqi Wang, Lin Chen, Boqun Xu, Shuo Wang, Peng Zhao, Xuan Qu, Ning Shen, Jing Wu, Xiang Lu, Guiping Wan, Bin Xue

**Affiliations:** 1 Translational Medicine Research Center, Children’s Hospital of Nanjing Medical University, Nanjing, Jiangsu, China; 2 Department of Geriatric Medicine, Jiangnan University Medical Center (Wuxi No.2 People’s Hospital), Jiangnan University, Wuxi, Jiangsu, China; 3 Department of Geriatric Medicine, Sir Run Run Hospital, Nanjing Medical University, Nanjing, Jiangsu, China; 4 Department of Gynecology and Obstetrics Medicine, Guangzhou First People’s Hospital, Guangzhou, Guangdong, China; 5 Department of Gynecology and Obstetrics Medicine, The Second Affiliated Hospital, Nanjing Medical University, Nanjing, Jiangsu, China; 6 China Exposomics Institute (CEI) Precision Medicine Co. Ltd, Shanghai, China; 7 Affiliated Hospital of Integrated Traditional Chinese and Western Medicine, Nanjing University of Chinese Medicine, Nanjing, Jiangsu, China; 8 Jiangsu Province Academy of Traditional Chinese Medicine, Nanjing, Jiangsu, China; 9 Department of General Surgery, The Affiliated Changzhou Second People’s Hospital of Nanjing Medical University, Changzhou, Jiangsu, China

**Keywords:** missed miscarriage, urinary xenoestrogens levels, plasticizers exposure, phytoestrogens, bisphenol A

## Abstract

**Introduction:**

Environmental exposure to hormone-like compounds that mimic endogenous estrogens is widespread. Xenoestrogens, a class of endocrine-disrupting chemicals, may interfere with endocrine function and adversely affect pregnancy outcomes. However, their association with missed miscarriage remains unclear. This study investigated the association between urinary xenoestrogen concentrations and the risk of missed miscarriage.

**Methods:**

This case–control study included 75 women with missed miscarriage and 75 women with normal pregnancies in Nanjing, China. Urinary concentrations of xenoestrogens were measured using liquid chromatography–tandem mass spectrometry. Associations between lifestyle factors and urinary xenoestrogen concentrations were assessed using Kendall’s tau-b correlation. Multivariable logistic regression was used to estimate odds ratios (ORs) and 95% confidence intervals (CIs) for missed miscarriage per interquartile range (IQR) increase in log-transformed urinary xenoestrogen concentrations. Receiver operating characteristic analysis was performed to evaluate the discriminatory performance of urinary xenoestrogen concentrations.

**Results:**

Urinary concentrations of five xenoestrogens were significantly higher in women with missed miscarriage than in controls. Bottled water consumption, cosmetic use, and beverage intake were positively correlated with the urinary concentrations of certain plasticizers. Higher urinary xenoestrogen concentrations were significantly associated with an increased risk of missed miscarriage. The areas under the curve ranged from 0.854 to 0.941, indicating good discriminatory performance.

**Conclusion:**

These findings suggest that detection of xenoestrogen levels may provide preliminary evidence of a potential association between environmental endocrine-disrupting exposure and missed miscarriage.

## 1 Introduction

Missed miscarriage is a specific type of spontaneous miscarriage in which the embryo or fetus has died, but the retained intrauterine products of conception have not expelled naturally before the completion of the 12th week of pregnancy (1). Miscarriage occurs in 8–15% of clinically recognized pregnancies and in approximately 30% of all pregnancies, and it is commonly diagnosed by ultrasonography (2). Missed miscarriage was undoubtedly a huge physical and psychological setback for women of childbearing age. The causes for missed miscarriage are complex and multifactorial, encompassing genetic factors (3), psychological influences (1), immune dysfunction (4), reproductive organs abnormalities (5), environmental chemicals (6), infections (7), and endocrine factors (8). Due to the limitations of clinical detection methods, we were unable to identify the causes of early missed miscarriages in a timely manner. Consequently, it is crucial to investigate the potential causes of missed miscarriage, as this is significant for identifying risk factors that can be utilized for early screening.

Recently, an increasing number of researchers have focused on the endocrine disruptors associated with missed miscarriage. Endocrine disruptors possess molecular structures that enable them to interact with hormone receptors, particularly estrogen and androgen receptors (9, 10). In contemporary society, our environment is increasingly laden with hormone-like agents that can mimic the action of endogenous estrogens. Xenoestrogens are classified as endocrine-disrupting chemicals that have the potential to disrupt the normal functioning of the endocrine system (11, 12). Xenoestrogens mimic or block the effects of natural estrogen by competitively binding to estrogen receptors in the body. This condition may trigger a range of physiological reactions, including affecting the normal function of the reproductive system and hormonal balance.

In this study, we focused on the most prevalent xenoestrogens encountered in everyday life. This heterogeneous group includes plasticizers (phthalates as mono-(2-ethylhexyl) phthalate (MEHP), mono-n-butyl phthalate (MBP) and phenols as bisphenol A (BPA)) and phytoestrogens (mycoestrogenes as zearalenone (ZEN) and isoflavone as Genistein (GEN). Evidence shows that these compounds have endocrine-disrupting effects and may impair female reproductive health. MBP exposure has been associated with abnormal ovarian and mammary gland development (13–15), while higher BPA levels have been observed in infertile women (16), suggesting potential adverse effects on fertility. ZEN, a fungal-derived estrogenic toxin, can disrupt endocrine function and impair ovarian development, leading to depletion of primordial follicles and increased risk of infertility (17, 18). GEN, a plant-derived phytoestrogen commonly found in soy products, may increase the risk of miscarriage and pregnancy complications due to its estrogen-like activity (19, 20). However, the relationship between xenoestrogen levels and missed miscarriages remains poorly understood, particularly in urine samples.

Therefore, to examine the association between urinary xenoestrogen levels and missed miscarriage, we conducted a case–control study that investigated the levels of five xenoestrogens in the urine of pregnant women. Additionally, we administered a questionnaire survey to identify potential sources of exposure to these endocrine disruptors among the participants. Furthermore, we employed a receiver operating characteristic curve analysis to further assess whether xenoestrogen has the potential to serve as a predictor of missed miscarriage risk.

## 2 Materials and methods

### 2.1 Study population and sample collection

A total of 75 cases and 75 controls were collected from the affiliated hospital of Integrated Traditional Chinese and Western Medicine. All subjects signed informed consent forms before participating. The cases included in the study were diagnosed as missed miscarriage. The inclusion criteria were as follows: (1) women aged 18–45 years; (2) gestational age ≤12 weeks based on the last menstrual period; (3) diagnosis of missed miscarriage according to ultrasonographic criteria, including crown-rump length ≥7 mm without fetal cardiac activity, mean gestational sac diameter ≥25 mm without an embryo, absence of an embryo with cardiac activity ≥2 weeks after a scan showing a gestational sac without a yolk sac, or absence of an embryo with cardiac activity ≥11 days after a scan showing a gestational sac with a yolk sac; (4) willingness to provide a urine sample and written informed consent. The exclusion criteria were as follows: (1) ectopic pregnancy, hydatidiform mole, biochemical pregnancy, or induced abortion; (2) known fetal chromosomal abnormalities or parental chromosomal abnormalities; (3) genital tract infection, active pelvic inflammatory disease, or other clinically diagnosed reproductive tract infections; (4) uterine malformations or other structural reproductive tract abnormalities; (5) history of assisted reproductive technology for the current pregnancy; (6) known exposure to medications or treatments that may affect early pregnancy outcomes; (7) incomplete clinical information or inability to provide a urine sample. The controls consisted of pregnant women with normal pregnancies and embryo development, as diagnosed by ultrasound at the obstetrical clinic of the sample hospital. All study participants were screened to include only women with less than 12 weeks of gestational age, a singleton pregnancy, and no other underlying health conditions.

Each subject finished a questionnaire survey which included subjects’ information (e.g., age, BMI, gestational age, education level, plastic bottled water use, cosmetics use, alcohol consumption history, etc.). All questionnaires were collected by trained interviewers. Follow-up results showed that all pregnant women in the normal group gave birth successfully. To ensure standardized urine collection, samples were obtained after diagnosis during hospitalization. Participants were instructed to fast and abstain from water intake after 10:00 p.m. the night before collection. The urine sample (second portion) was collected at approximately 8:00 a.m. the following morning using plastic Vacuette® Urine Collection Cups (Greiner Bio-One International AG, Austria). All samples were stored at −20 °C until analysis.

### 2.2 Measurement of urinary pollutant metabolites

Samples were collected at predetermined time intervals and filtered through a membrane filter of 0.22 μm. The pH of respective mixtures was adjusted to 5.4 by adding acetic acid–sodium acetate buffer (0.5 M), then β-glucuronidase/arylsulfase (10 μL), and vitamin C (5 mg) were added and incubated overnight at room temperature to complete the enzymatic hydrolysis. The samples after enzymatic hydrolysis were extracted by solid-phase extraction with an SPE column (C18 ENVIJI 0.25 g). The extract was eluted with methanol (2 mL) and dried with nitrogen. Finally, the dried residue was reconstituted in methanol (100 μL), and the resulting solution was used for subsequent analysis. 50 μL of the analyte to be tested was transferred to a liquid chromatography bottle with a microsyringe, which was specially used for the injection analysis of GEN. Evaluation of GEN, ZEN, BPA, MBP, and MEHP levels in the urine of examinees was performed using Liquid chromatography-mass spectrometry (LC–MS). Quantification was done using an external standard calibration method with matrix-matched standards, showing good linearity across a concentration range. Standard working solutions of 1 μg/mL for GEN, BPA, MBP, and MEHP were prepared using methanol as the solvent. After continuous dilution of 10^4^ times, it became the standard working solution of 100 pg./mL. Take GEN as an example, different concentrations of GEN standard working solution were prepared. 50 μL of each GEN standard working solution was transferred to a liquid chromatography bottle with a microsyringe, which was specially used for the injection analysis of GEN standard samples.

### 2.3 Statistical analysis

The normality of the distribution was assessed using the Shapiro–Wilk test. Student’s t-test for two independent samples was used for data that conformed to a normal distribution. Skewed data were expressed as median and quartile ranges, and comparison between two independent groups were analyzed by Mann–Whitney *U* test. Pearson *χ*^2^ test was used to compare qualitative variables represented as frequencies. The correlations between lifestyle characteristics and urinary xenoestrogen concentrations were calculated using Kendall’s tau-b correlation coefficient. The correlation between missed miscarriage and log-transformed urinary xenoestrogen levels were calculated using separate binary logistic regression models for each xenoestrogen, adjusting for age, BMI, gestational age, education, plastic bottled water use, cosmetics use, and alcohol consumption history. A receiver operating characteristic (ROC) curve was plotted, and area under ROC curve calculated. All tests were two-sided, and a significance level of *p* < 0.05 was considered statistically significant. Statistical analysis was performed to use SPSS 23.0 software (IBM SPSS, Chicago, IL, USA). Graphs were generated by GraphPad Prism (GraphPad Prism 9.2, GraphPad Software, USA).

## 3 Results

### 3.1 Participant characteristics

A total of 150 patients were included in the study with 75 cases of missed miscarriage and 75 cases of continuing pregnancy to second trimester. The characteristics were listed in Table 1. The median age was 30 years (ranging from 27 to 33) in the case group and 31 years (ranging from 28 to 34) in the control group. There were no significant differences in age and gestational age between the control and case group (Z = 1.004, *p* = 0.315; Z = 0.626, *p* = 0.531). The results showed that the cases had slightly higher BMI and lower educational levels compared to controls (Z = 2.06, *p* = 0.039; *χ*^2^ = 15.79, *p* = 0.0004). The significant difference was observed in the use of plastic bottled water and cosmetics between cases and controls (*χ*^2^ = 53.16, *p* < 0.0001; *χ*^2^ = 33.89, *p* < 0.0001). Of note, 96% of missed miscarriage patients had a history of frequent alcohol drinking, compared to 62.7% in the control group (*χ*^2^ = 26.80, *p* < 0.0001).

**Table 1:** Characteristics of the study population.

Variables		Control (*n* = 75)	Case (*n* = 75)	Z/*χ*^2^	*P*^a^
		Median (25th, 75th)	Median (25th, 75th)		
Age (25th, 75th)		31 (28, 34)	30 (27, 33)	1.004	0.315
BMI^b^ (25th, 75th)		20.75 (19.59, 22.83)	22.04 (19.56, 24.03)	2.06	0.039
Gestational age (25th, 75th)		9.78 (8.87, 10.66)	9.96 (8.93, 10.82)	0.626	0.531
Education					
High school or below (%)		13 (17.30)	27 (36.00)	15.79	0.0004
Undergraduate (%)		46 (61.30)	46 (61.30)		
Graduate school or higher (%)		16 (21.30)	2 (2.70)		
Plastic bottled water use					
Never (%)		29 (38.70)	10 (13.30)	53.16	<0.0001
Occasional (%)		40 (53.30)	16 (21.30)		
Frequent (%)		6 (8.00)	49 (65.30)		
Cosmetics use					
Never (%)		32 (42.70)	15 (20.00)	33.89	<0.0001
Occasional (%)		36 (48.00)	20 (26.70)		
Frequent (%)		7 (9.30)	40 (53.30)		
Drinking history					
Never (%)		1 (1.30)	1 (1.30)		
Occasional (%)		27 (36.00)	2 (2.70)	26.80	<0.0001
Frequent (%)		47 (62.70)	72 (96.00)		

a The different demographic characteristics between control and case were compared using Mann–Whitney test and Chi-squared test.

b BMI, body mass index.

### 3.2 Xenoestrogen concentrations in urine samples

Table 2 presented the xenoestrogen concentrations in the urine of 150 subjects with MBP showing the highest level followed by MEHP, GEN, BPA, and ZEN. The median concentrations of BPA, MBP, MEHP, GEN, ZEN were 2.27 μg/g, 56.88 μg/g, 3.30 μg/g, 3.23 μg/g, 1.39 ng/g, respectively. Additionally, Table 3 displayed the differences of xenoestrogen concentrations between the case and control group. The results indicated that the case group exhibited higher concentrations of GEN (Z = −7.493, *p* < 0.0001), ZEN (*Z* = −8.342, *p* < 0.0001), BPA (Z = −9.324, *p* < 0.0001), MBP (Z = −8.903, *p* < 0.0001), and MEHP (Z = −8.542, *p* < 0.0001) compared to the control group (Figure 1).

**Table 2:** Urinary xenoestrogen concentrations (/urine creatinine) in urine samples.

Pollutants concentration	Total (*n* = 150)				
	Median	Q1^a^	Q2	Q3	Q4
GEN (μg/g)	3.23	<1.77	1.77 ~ 3.23	3.23 ~ 5.29	>5.29
ZEN (ng/g)	1.39	<0.56	0.56 ~ 1.39	1.39 ~ 2.42	>2.42
BPA (μg/g)	2.27	<0.88	0.88 ~ 2.27	2.27 ~ 4.47	>4.47
MBP (μg/g)	56.88	<25.42	25.42 ~ 56.88	56.88 ~ 99.66	>99.66
MEHP (μg/g)	3.30	<1.67	1.67 ~ 3.30	3.30 ~ 5.73	>5.73

a Q: quartile.

**Table 3:** Urinary xenoestrogen concentrations (/urine creatinine) in control group and case group.

Pollutants	Control (*n* = 75)	Case (*n* = 75)	Z	*P*^b^
	Median (*IQR*^a^)	Median (*IQR*^a^)		
GEN (μg/g)	1.90 (1.12, 3.05)	4.70 (3.68, 7.25)	−7.493	<0.0001
ZEN (ng/g)	0.58 (0.36, 1.07)	2.23 (1.67, 3.65)	−8.342	<0.0001
BPA (μg/g)	0.88 (0.63, 1.53)	4.38 (3.15, 6.99)	−9.324	<0.0001
MBP (μg/g)	25.52 (18.22, 47.77)	89.21 (70.55, 134.12)	−8.903	<0.0001
MEHP (μg/g)	1.67 (1.15, 2.81)	5.54 (3.61, 7.87)	−8.542	<0.0001

a IQR, Inter-quartile range.

b The Mann–Whitney test was used to compare the differences between controls and cases.

**Figure 1:**
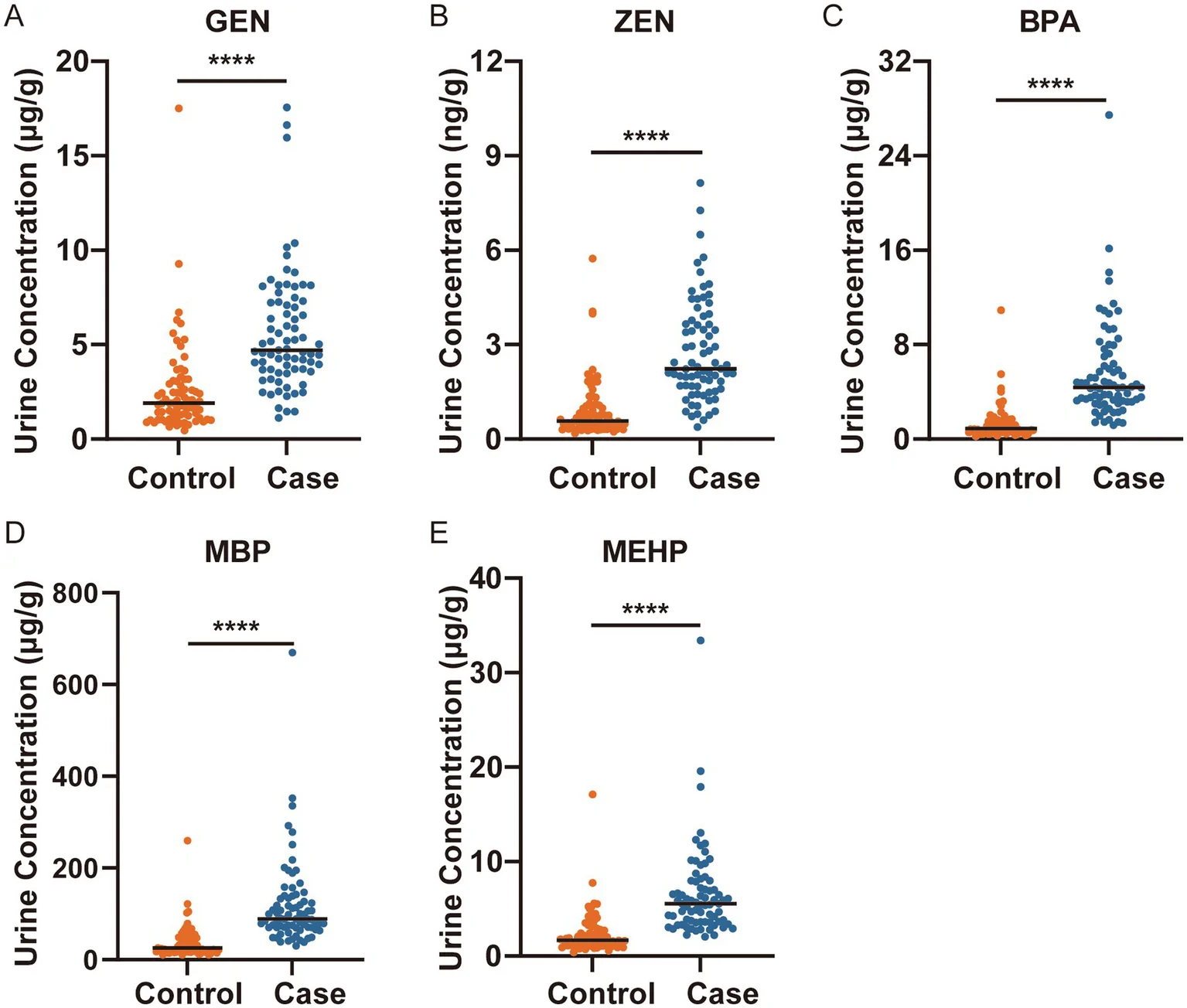
Xenoestrogens concentrations in urine samples between the control and case group. **A–E**, the urinary concentration of GEN, ZEN, BPA, MBP, MEHP, respectively. ***p <0.0001.

### 3.3 Correlation between lifestyle characteristics and urinary plasticizer concentration

Among the five xenoestrogen indexes studied, three (BPA, MBP, MEHP) are commonly originated from plastic products. To investigate the potential correlation between plasticizer content in human body and the daily use of plastic products, an analysis of urine levels of plasticizers and lifestyle characteristics was conducted. The analysis showed that the use of plastic bottled water was positively correlated with the concentrations of BPA (*r* = 0.258, *p* = 0.0001), MBP (*r* = 0.281, *p* < 0.0001), and MEHP (*r* = 0.248, *p* = 0.0001) based on Kendall’s Tau-b correlation coefficients. Moreover, the cosmetics use and alcohol consumption were also found to be positively associated with the levels of plasticizers, as shown in Table 4.

**Table 4:** Kendall’s Tau-b correlation coefficient between lifestyle characteristics and urinary xenoestrogen concentrations.

Variables	BPA		MBP		MEHP	
	*r*	*p*	*r*	*p*	*r*	*p*
Plastic bottled water use	0.258	0.0001	0.281	<0.0001	0.248	0.0001
Cosmetics use	0.224	0.0004	0.255	0.0001	0.241	0.0001
Drinking history	0.261	0.0001	0.242	0.0003	0.249	0.0002

### 3.4 Potential risk factors for missed miscarriage

To further investigate the relationship between xenoestrogen levels and the incidence of missed miscarriage, a binary logistic regression analysis was performed. Table 5 presents the logistic regression models evaluating the associations between urinary xenoestrogen concentrations and missed miscarriage after adjustment for age, BMI, gestational age, education, plastic bottled water use, cosmetics use, and alcohol consumption history. Considering the skewed distributions and different measurement scales of urinary xenoestrogens, all exposure concentrations were log-transformed, and ORs were estimated per interquartile range (IQR) increase in the log-transformed concentration of each compound.

**Table 5:** Associations of urinary xenoestrogen concentrations with missed miscarriage.

Variables	B	OR	95% CI		*p*
			Lower	Upper	
GEN	0.426	1.532	1.223	1.918	<0.001
ZEN	1.080	2.945	1.767	4.909	<0.001
BPA	1.410	4.096	2.330	7.200	<0.001
MBP	0.049	1.050	1.029	1.071	<0.001
MEHP	0.733	2.082	1.520	2.851	<0.001

Age, BMI, gestational age, education, plastic bottled water use, cosmetics use, and drinking history were adjusted.

After multivariable adjustment, each IQR increase in log-transformed urinary GEN and ZEN concentrations was significantly associated with increased odds of missed miscarriage. In addition, higher log-transformed urinary concentrations of plasticizer-related compounds, including BPA, MBP, and MEHP, were also significantly associated with increased odds of missed miscarriage. The detailed ORs and 95% CIs are shown in Table 5. In logistic regression analyses, ORs were estimated per IQR increase in log-transformed urinary xenoestrogen concentrations. Higher levels of urinary xenoestrogens were associated with increased odds of missed miscarriage.

### 3.5 Predictive performance of urinary xenoestrogen concentrations in missed miscarriage

To evaluate the potential predictors of missed miscarriage, a ROC curve analysis was introduced, as depicted in Figure 2. The area under the curve (AUC) for each xenoestrogen index is detailed in Table 6. Notably, BPA exhibited the best predictive performance for missed miscarriage of which an AUC was 0.941 (95% CI: 0.902–0.979, *p* < 0.0001), followed by MBP (AUC = 0.921, 95% CI: 0.877–0.965, *p* < 0.0001), MEHP (AUC = 0.904, 95% CI: 0.856–0.952, *p* < 0.0001), ZEN (AUC = 0.895, 95% CI: 0.843–0.947, *p* < 0.0001), and GEN (AUC = 0.854, 95% CI: 0.792–0.916, *p* < 0.0001). These findings suggest that the xenoestrogen index demonstrates promising accuracy (AUC > 0.85) in predicting missed miscarriage.

**Figure 2:**
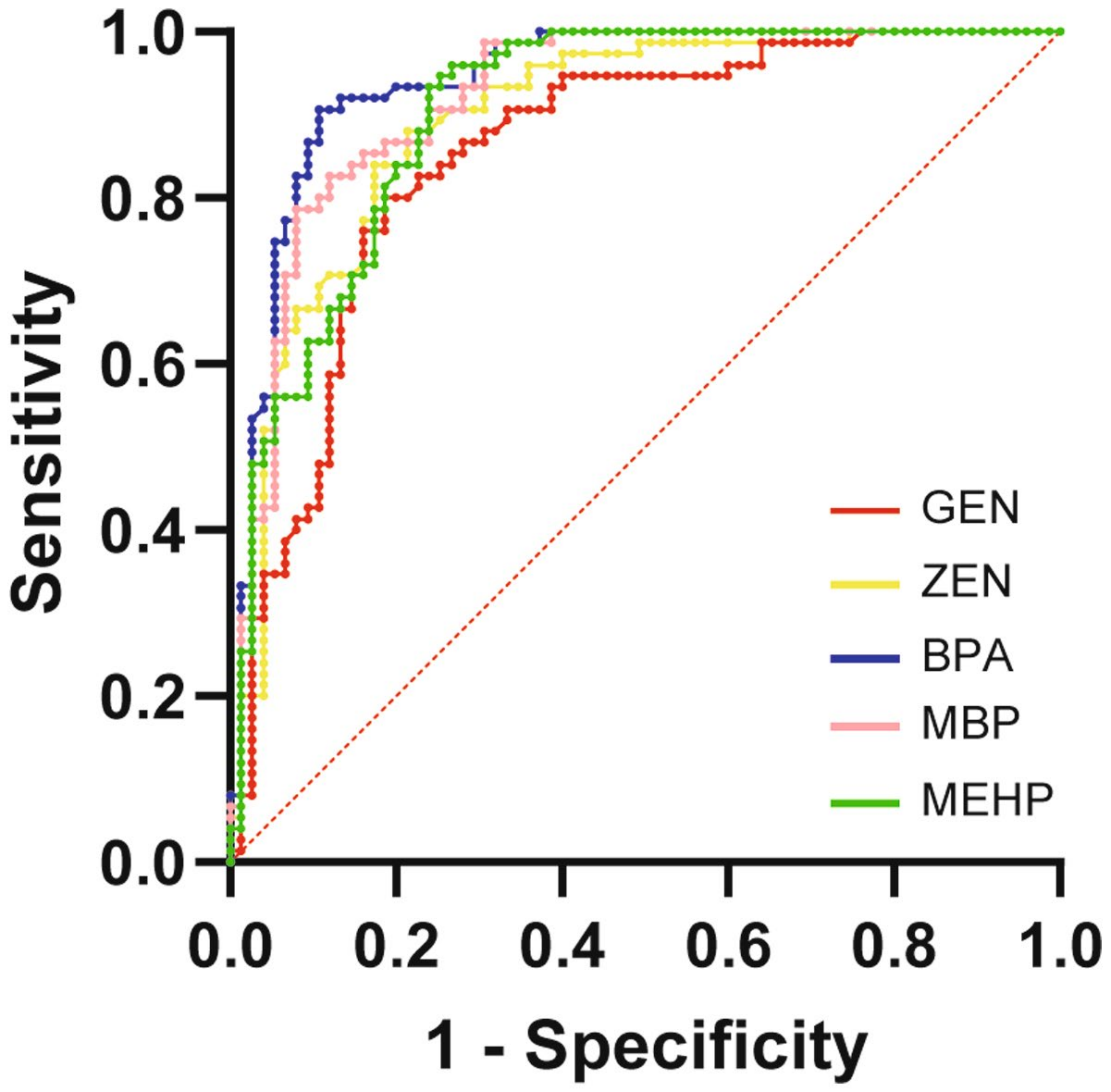
ROC curve analysis for missed miscarriage.

**Table 6:** The AUC value of ROC curve.

Variables	Area	95% CI		*p*
		Lower	Upper	
GEN	0.854	0.792	0.916	<0.0001
ZEN	0.895	0.843	0.947	<0.0001
BPA	0.941	0.902	0.979	<0.0001
MBP	0.921	0.877	0.965	<0.0001
MEHP	0.904	0.856	0.952	<0.0001

## 4 Discussion

In this case–control study, higher urinary concentrations of xenoestrogens were associated with increased odds of missed miscarriage after adjustment for potential confounders. The associations were observed for both phytoestrogen and plasticizer related compounds, indicating that internal exposure to xenoestrogenic chemicals may be relevant to the occurrence of missed miscarriage. In addition, lifestyle-related factors, particularly the use of plastic bottled water and cosmetics, were associated with urinary xenoestrogen levels, suggesting that daily consumer product use may contribute to xenoestrogen exposure. These findings may have biological relevance because xenoestrogens can mimic or interfere with endogenous estrogen activity and may disrupt the hormonal balance required for implantation, placental development, and early pregnancy maintenance. Previous studies have also linked exposure to plastic additives and other endocrine-disrupting chemicals with adverse reproductive and pregnancy outcomes, including miscarriage. Our findings therefore support the hypothesis that environmental exposure to xenoestrogenic compounds may be involved in missed miscarriage.

Research shows that changes in hormone levels during different stages of pregnancy may have an important impact on pregnancy outcomes. Estrogen is an important class of sex hormones that are critical to the development and function of the female reproductive system (21). During pregnancy, estrogen levels increase significantly to support the growth and development of the fetus. However, high estrogen levels may also be associated with an increased risk of miscarriage. Pregnant women may be unintentionally exposed to excessive estrogens from the environment, including plasticizers and phytoestrogens. BPA and phthalates are chemicals widely used in industry, especially in plastic manufacturing (22, 23). The result of our study showed that the median concentration of phthalate metabolite-MBP was the highest, which consistent with the studies in Anhui and Wuhan pregnant women, while they reported median concentrations of MBP as 52.60 and 78.80 ng/mL (23, 24). Our research indicates that the concentrations of BPA, MBP, and MEHP in the missed miscarriage group were significantly higher than those in the control group (*p* < 0.01, *p* < 0.01, *p* < 0.01, respectively). Although urinary BPA and phthalate metabolites are established biomarkers of exposure rather than disease-specific biomarkers (25), their measurement provides a non-invasive approach to assess internal exposure to endocrine-disrupting chemicals. Accordingly, urinary xenoestrogen levels in this study may be considered potential exposure-related biomarkers associated with missed miscarriage, but not validated diagnostic or predictive biomarkers. The lifestyle questionnaire completed by the participants revealed that women in the case group consumed more plastic bottled water than those in the control group (*p* < 0.001). Furthermore, the use of plastic bottled water during pregnancy was positively associated with BPA concentrations (*r* = 0.258, *p* < 0.001), MBP concentrations (*r* = 0.281, *p* < 0.001), and MEHP concentrations (*r* = 0.248, *p* < 0.001). Moreover, the use of cosmetics and consumption of drink were found to be positively associated with the levels of plasticizers in our study. These findings may help to explain why women appeared to have more opportunity exposure to phthalates.

Considering the rise in missed miscarriage incidence, changes in diet have been considered as a potential cause. Phytoestrogens are plant-derived and food-related compounds known to have estrogenic activity. The most prominent phytoestrogens are GEN (isoflavones) and ZEN (mycoestrogens). Our study demonstrated that the concentrations of GEN and ZEN in the case group were significantly higher than those in the control group. Likewise, a binary logistic regression analysis revealed a positive correlation between the Log-transformed concentrations of GEN and ZEN in urine and the onset of missed miscarriage. The ROC curves indicate that these compounds could effectively predict the occurrence of missed miscarriages. Additionally, extensive work with human breast cancer cell lines has shown estrogenic stimulation in those possessing ER but a reduction in DMBA-induced breast cancers in rodents given ZEN (26). However, certain flavonoids, such as total flavonoids from semen cuscutae are widely used to treat diseases related to the female reproductive system in clinical including renal infertility, impotence, endometriosis, polycystic ovary syndrome, and menstrual disorders, etc (27, 28). These findings are relevant to missed miscarriage because urinary xenoestrogen concentrations may reflect internal exposure to endocrine-disrupting compounds. Early pregnancy maintenance depends on tightly regulated hormonal signaling, implantation, decidualization, placental development, and embryonic viability. Xenoestrogenic compounds may interfere with these processes by mimicking or antagonizing endogenous estrogen activity and disrupting hormonal homeostasis. Therefore, the observed associations between urinary xenoestrogen levels and missed miscarriage are biologically plausible and support further investigation of these compounds as exposure-related biomarkers. Because urine collection is non-invasive, urinary xenoestrogens may have potential value for exploratory early risk assessment. However, the present case–control study can only identify associations between cases and controls and cannot establish causality or validated predictive performance. Thus, the ROC findings should be interpreted as preliminary and hypothesis-generating. Further prospective studies with repeated exposure measurements and external validation are necessary before urinary xenoestrogens can be considered clinically useful biomarkers for missed miscarriage risk assessment. Although there are few reports on the reproductive toxicity mechanisms of xenoestrogens, the existing research provides a basis for further investigation into missed miscarriages. This study has several limitations. Firstly, selecting cases from the hospital may exclude some pregnant women who experience a miscarriage without seeking medical attention, potentially introducing selection bias. Secondly, we collected only a single spot urine sample from each participant which may miss the variation of the urine concentrations of the xenoestrogens. However, previous studies have indicated that a single spot urine sample can moderately reflect long-term exposure to phthalates (29). Potential contamination from urine collection containers cannot be fully excluded; however, identical procedures were applied across groups, suggesting any effect would be non-differential. Finally, given that this is a single-center study with a relatively limited sample size, larger prospective studies with external validation are needed to determine whether xenoestrogen exposure has predictive or mechanistic relevance in missed miscarriage.

## 5 Conclusion

In summary, our research results indicate that levels of xenoestrogens in the urine of patients experiencing missed miscarriage are elevated, and these levels are positively correlated with the incidence of missed miscarriage. Furthermore, monitoring xenoestrogens levels in urine can effectively track the occurrence of missed miscarriage, providing a valuable detection method for early prevention. This study also establishes a baseline for future research and offers a scientific foundation for regulatory authorities to control the use of plasticizers and phytoestrogens.

## Data Availability

The original contributions presented in the study are included in the article/supplementary material, further inquiries can be directed to the corresponding author.
